# A systematic comparison of cavitation regimes and histotripsy efficiency across pulse duration and repetition rate in a fibrous tissue mimicking phantom

**DOI:** 10.21203/rs.3.rs-9717731/v1

**Published:** 2026-06-03

**Authors:** Yashwanth Nanda Kumar, Yak-Nam Wang, Wayne Kreider, Qinghua Han, Jonathan T.C. Liu, Eli Vlaisavljevich, George R. Schade, Adam D. Maxwell

**Affiliations:** 1 University of Washington, Center for Industrial and Medical Ultrasound, Seattle, 98105, United States of America; 2 University of Washington, Department of Bioengineering, Seattle, 98195, United States of America; 3 University of Washington, Department of Mechanical Engineering, Seattle, 98195, United States of America; 4 Stanford University, Department of Bioengineering, Stanford, 94305, United States of America; 5 Stanford University, Department of Pathology, Stanford, 94304, United States of America; 6 University of Washington, Department of Laboratory Medicine and Pathology, Seattle, 98195, United States of America; 7 Virginia Polytechnic Institute and State University, Department of Biomedical Engineering and Mechanics, Blacksburg 24061, United States of America; 8 University of Washington, Department of Urology, Seattle, 98195, United States of America

**Keywords:** Fibrous tissue-mimicking phantom, cavitation dynamics, histotripsy regimes, pulse structure effects, complete liquefaction, open-top light-sheet microscopy

## Abstract

Histotripsy is a focused ultrasound technique that mechanically liquefies tissue through cavitation bubble activity and has shown promise for noninvasive tumor ablation. However, treating fibrous, mechanically robust tissues remains challenging, and the influence of pulse parameters on cavitation dynamics and treatment efficiency in these tissues is not well understood. Here, we systematically investigated the combined effects of pulse duration and pulse repetition frequency (PRF) on cavitation behavior and lesion formation in a fibrous tissue-mimicking double-network hydrogel. Pulse duration and PRF were varied over four orders of magnitude while maintaining a constant 1% duty cycle. High-speed photographic imaging, shear wave elastography (SWE), B-mode ultrasound, and open-top light-sheet (OTLS) microscopy were used to relate cavitation behavior to phantom damage and liquefaction. Short-pulse, high-PRF exposures (1–10 cycles) produced larger bubble cloud areas, higher frame-to-frame correlation, and greater spatial persistence, whereas longer-pulse, low-PRF exposures (≥100 cycles) produced more localized cavitation and contiguous lesion formation. Among the tested parameters, 1,000 -cycle pulses at 10 Hz yielded the highest volumetric liquefaction rates. In contrast, short-pulse, high-PRF conditions generated substantial total damage but little to no fully liquefied volume. These findings show that pulse duration and PRF strongly govern histotripsy outcomes in fibrous tissues

## Introduction:

Histotripsy is a non-invasive focused ultrasound modality that mechanically ablates tissue by generating and exciting cavitation bubbles^1,2^. Unlike thermal high-intensity focused ultrasound, which relies primarily on heat deposition and coagulative necrosis, histotripsy fractionates tissue through cavitation-mediated stresses. The rapid expansion and collapse of these bubbles induce strain on the surrounding tissue, causing breakdown and liquefaction. This mechanism is attractive because it can enable targeted tissue liquefaction while limiting thermal injury to adjacent structures, particularly when the duty cycle is kept low compared with traditional thermal HIFU procedures.

Currently, multiple modalities for nucleation of histotripsy bubbles have been developed. Intrinsic threshold histotripsy generates inertial cavitation through the negative pressure phase of 1–2 cycle pulses^3,4^. It is often employed to create precise lesions, as the pressure threshold for nucleation has been demonstrated to be consistent and predictable between many tissue types. Shock-scattering histotripsy utilizes multi-cycle pulses (typically 3 – 50 cycles), in which the negative pressure phase of the initial cycles causes intrinsic nuclei within the focal zone to expand. Once these bubbles undergo inertial cavitation, they scatter the compressive shocks of the incoming pulse train, inverting their polarity to create a higher tensile wave. The subsequent constructive interference creates a dense bubble cloud that grows toward the transducer face and its interaction with the tissue causes mechanical disintegration^5^. Boiling histotripsy uses milliseconds-long pulses to generate millimeter-sized boiling bubbles^6–8^. Shock-induced heating leads to a rapid temperature increase, where the combination of negative pressure and elevated temperature leads to bubble nucleation and rectified growth^9,10^. Interaction of the enlarged bubbles with incident pulses results in the formation of shock-scattered clouds that expand toward the transducer^11^. Secondary boiling bubbles may appear distal to the primary boiling bubble, and cavitation may appear proximal to the boiling bubbles to collectively contribute to the liquefaction process. Although these pulses last several milliseconds and involve the formation of a boiling bubble, the tissue ablation process remains predominantly mechanical due to the low duty cycle (≤1%) used. Lastly, other forms of histotripsy have been explored with pulse durations between the three types listed above (i.e. >100 cycles to <10,000 cycles), and their mechanisms of nucleation may include one or more of the processes described above^12,13^.

Tissues with higher stiffness and toughness are resilient to histotripsy and often require a higher number of pulses to achieve disintegration for a given modality^14,15^. This challenge is particularly relevant for fibrotic pathologies, where successful preclinical treatment does not necessarily translate into consistent clinical benefit. For example, following a successful pre-clinical study^16^, a clinical trial using histotripsy to treat human benign prostatic hyperplasia by a transperineal approach did not achieve objective improvements in symptoms^17^. More broadly, various histotripsy modalities have been investigated for fibrous tissues in *ex vivo* studies, including uterine fibroids^18,19^, cholangiocarcinoma^20^, prostate cancer^21^, human BPH^22–24^, and bladder wall^25^. The variations in devices, exposure parameters, and conditions between studies make direct comparisons challenging and complicate the determination of which parameters have the most significant impact on effectively treating a specific tissue type. However, comparisons within individual studies have provided important insight. For instance, our previous study employing different parameters in ex-vivo human BPH tissue found marked increase in treatment efficiency using longer pulses delivered at low PRF (20 cycles, 10 Hz PRF) compared to short pulses delivered at high PRF (3 cycles, 500 Hz PRF)^22^. This suggested that histotripsy outcomes in fibrous tissue can be improved through parameter optimization, but the cavitation behavior underlying these differences remained unclear. Because treatment time is a major factor in clinical feasibility, further optimization of histotripsy for liquefying fibrous tissue is particularly important for debulking applications.

Past research focused on understanding the mechanisms of ablation by studying the bubble cloud characteristics in RBC agarose phantoms^26–31^, and polyacrylamide phantoms^32,33^. In a previous study^34^, we noted that while these phantoms can be made stiff, they do not accurately represent tough fibrous tissue or its response to histotripsy, as they have a relatively low toughness. We introduced and validated a double-network hydrogel as a closer surrogate for tough connective tissues found in many pathologic conditions. Based on these previous developments^22,34,35^, the present study aimed to understand the ablation efficacy of different parameters sets spanning histotripsy modalities in a fibrous-tissue mimicking double-network phantoms, delivered by a single transducer at a constant duty cycle and multiple doses, to evaluate how pulse sequence influenced liquefaction outcomes. High-speed photography was further used to characterize bubble dynamics and relate them to treatment outcome. We hypothesized that the tested parameter sets would identify clear trends in ablation correlated with distinct cavitation behaviors, providing insight into strategies for treating fibrous tissues.

## Results:

### Study design overview

To evaluate how pulse timing influences histotripsy efficiency in fibrous media, five pulse-parameter sets were tested in a double-network hydrogel phantom while maintaining a constant 1% duty cycle using a focused 1MHz transducer (f# 0.72). Pulse duration and PRF were varied inversely, from 1-cycle pulses at 10,000 Hz to 10,000 -cycle pulses at 1 Hz. Outcomes were assessed at single focal points and in 3 × 3 multi-point treatments. Cavitation behavior was quantified using high-speed imaging, while treatment response was evaluated using phase contrast microscopy, B-mode ultrasound, shear wave elastography, and open-top light-sheet microscopy (OTLS). This structure allowed cavitation behavior to be compared directly with partial damage, fully liquefied volume, and treatment rate.

### Acoustic output and treatment parameters

The transducer acoustic output was characterized across parameter sets to confirm that exposures were delivered under comparable relative driving conditions. The linear −6 dB beamwidth of the transducer was 9.8 mm axially and 1.5 mm laterally, providing the geometric basis for the single-point exposures and the 0.75 mm spacing used for 50% overlap in multi-point treatments. At representative treatment amplitudes, nonlinear simulations estimated narrower effective beamwidths based on the compressive peak pressure, with axial and lateral widths of 3.4 mm and 0.5 mm, respectively, and broader dimensions based on the tensile pressure field, with axial and lateral widths of 8.4 mm and 1.8 mm, respectively.

For treatments using 10–10,000 cycles, acoustic outputs were set to approximately 20% above the cavitation threshold for each condition, as determined from the onset of a consistent hyperechoic cavitation cloud on B-mode imaging. These conditions resulted in measured peak positive pressures of P+ = 85.2–114.3 MPa and peak negative pressures of P− = 15.8–17.0 MPa based on FOPH measurements. Corresponding nonlinear simulations yielded similar values, with P+ = 91.3–110.2 MPa and P− = 16.3–17.4 MPa. The 10-cycle condition required the highest absolute drive level within the multi-cycle treatments because of its elevated cavitation threshold, resulting in P+ = 114.3 MPa and P− = 17.0 MPa.

For single-cycle treatments, exposures were conducted at a higher input source voltage of 240 V because the finite bandwidth of the transducer reduced the first-cycle output relative to the steady-state waveform. Since this voltage exceeded the hydrophone measurement range, calibrated HIFU beam simulations were used to estimate *in situ* pressure, yielding P+ ≈ 40.0 MPa and P− ≈ 13.6 MPa. These values are consistent in trend with prior single-cycle histotripsy studies conducted at lower PRFs (e.g., 1,000 Hz), where cavitation thresholds of P− ≈ 15 MPa have been reported^27^. The lower P+/P− ratio for the single-cycle condition reflects the reduced first-cycle amplitude and lower degree of nonlinear shock formation compared with the steady-state portion of the multi-cycle waveforms.

### Single-point and multi-point treatment workflow

Single-point exposures were used to relate cavitation behavior to lesion morphology and local mechanical changes. Multi-point treatments were then used to evaluate whether these trends persisted under volumetric ablation conditions and to estimate fully liquefied volume and treatment rate.

### Cavitation behavior during single-point exposures

**Figure 1.**
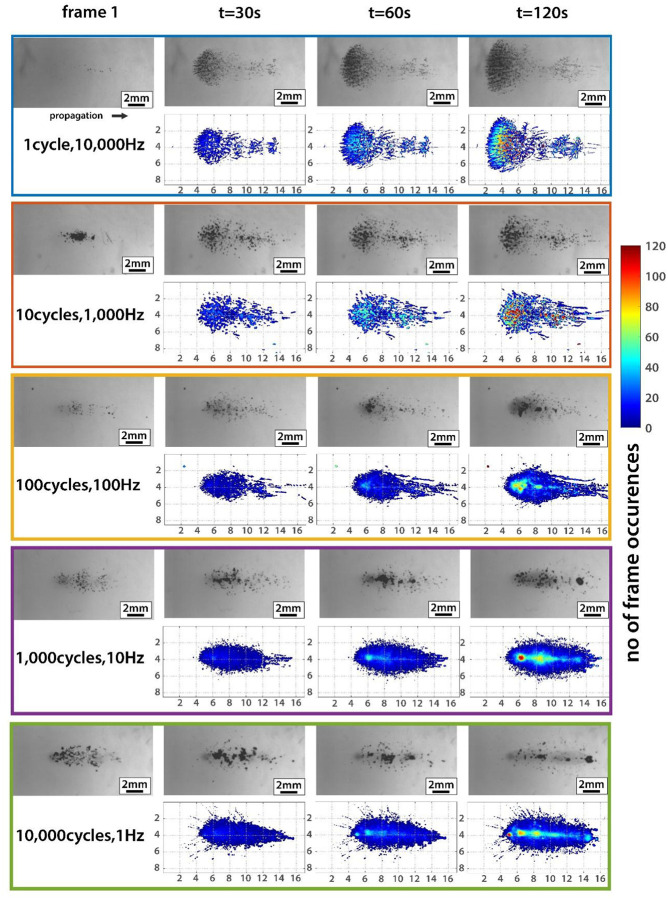
Representative high-speed camera image frames from different time points (time *t* in seconds) with cumulative cavitation maps below each photo. The transducer is positioned to the left of the frame and the acoustic beam traversing left to right. The cumulative maps show the bubble cloud’s spread and tracks the number of frames over which a bubble appears in each location.

Single-point exposures were first used to compare cavitation behavior across pulse parameters before evaluating lesion morphology and treatment efficiency. Representative high-speed camera images and cumulative cavitation maps are shown in Figure 1. Cavitation was observed for all parameter sets, but the spatial extent, persistence, and localization of the bubble clouds differed substantially with pulse duration and PRF.

**Figure 2.**
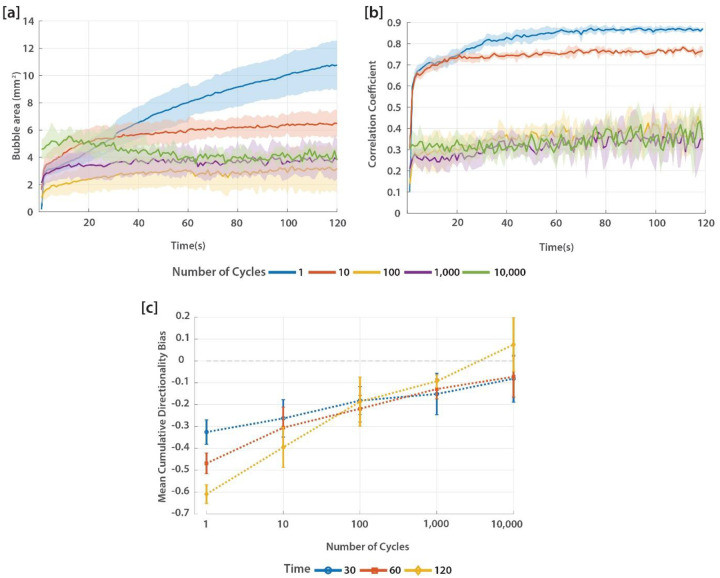
(a) Bubble cloud area as a function of treatment time for each pulse duration. (b) Pearson correlation coefficient between successive binarized cavitation frames. (c) Mean cumulative directionality bias calculated relative to the early-treatment cavitation centroid for 30, 60, and 120 s analysis windows. Negative values indicate prefocal accumulation toward the transducer, positive values indicate post-focal accumulation, and values near zero indicate a balanced cumulative cavitation distribution.

The temporal evolution of bubble cloud area is shown in Figure 2a. At early time points (t < 10s), bubble cloud sizes were comparable across all parameter sets, with no significant differences observed between conditions (one-way ANOVA, p > 0.05). With increasing treatment time, distinct behaviors emerged. The 1-cycle condition exhibited continuous growth in bubble area throughout the exposure and was significantly larger than all other conditions at 120 s (one-way ANOVA with Tukey post hoc, p < 0.05). In contrast, the ≥10-cycle conditions reached a quasi-steady state within approximately 20–30 s, with minimal subsequent growth and no significant differences among these conditions at 120s. Consistent with these trends, early-to-late comparison showed a significant increase in bubble area for the 1-cycle condition (paired comparison, p < 0.05), whereas the ≥10-cycle conditions did not show significant changes.

Cavitation persistence for each pixel in the image was assessed using the Pearson correlation coefficient between successive frames (Figure 2b). The 1-cycle and 10-cycle conditions exhibited higher correlation values throughout the exposure compared to ≥100 cycle conditions. The ≥100 cycle conditions showed lower and relatively stable correlation values over time. Comparison between early and late time points indicated an increase in correlation for the 1-cycle condition (paired comparison, p < 0.05), while no significant changes were observed for ≥10 cycle conditions.

Cumulative directionality bias was used to determine whether cavitation activity accumulated preferentially toward the transducer-facing or distal side of the early cavitation location (Figure 2c). Values represent the mean cumulative directionality bias across pooled video-window measurements within each pulse-duration condition. Negative values indicate prefocal accumulation toward the transducer, positive values indicate post-focal accumulation, and values near zero indicate a more balanced cumulative distribution. The 1-cycle condition showed the strongest negative bias, with the magnitude increasing from 30 to 120 s. The 10-cycle condition also showed negative bias values, although the magnitude was lower than that of the 1-cycle condition. The ≥100-cycle conditions showed values closer to zero across treatment durations, with the 1,000 - and 10,000 -cycle conditions approaching zero or slightly positive values at longer treatment durations.

### Lesion morphology after single-point exposures

**Figure 3.**
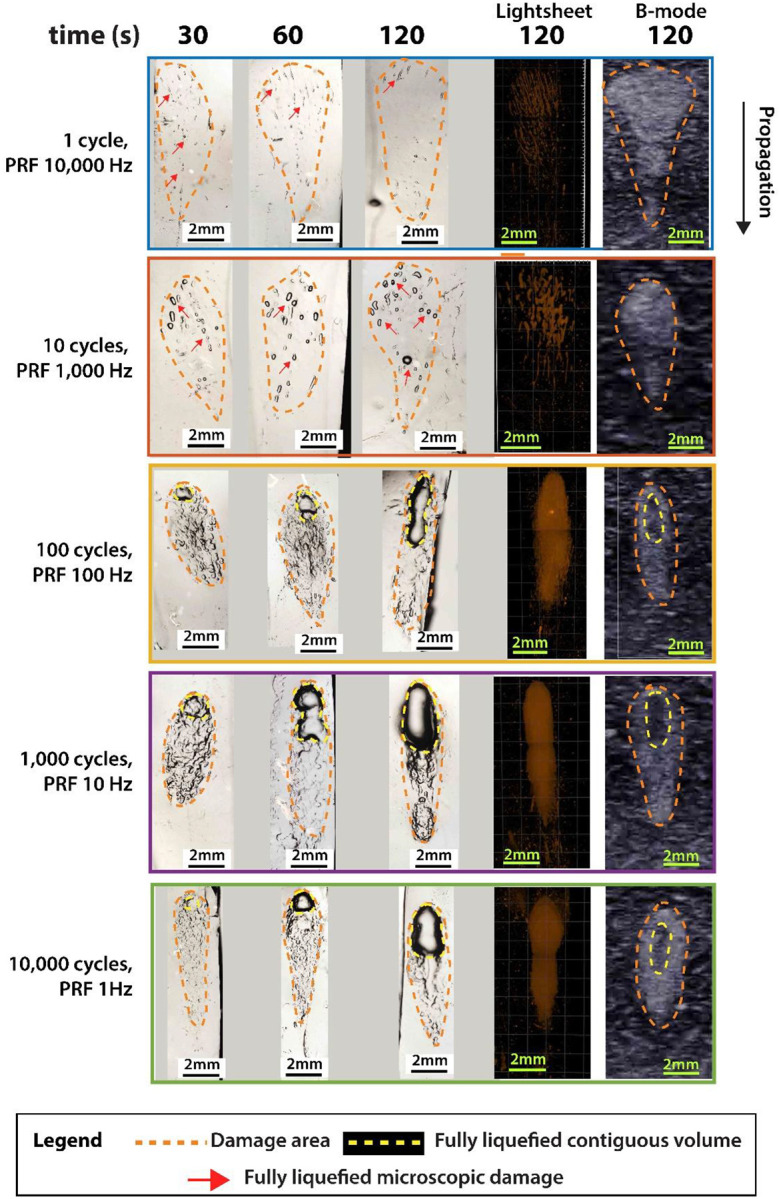
Phase contrast microscopy images and OTLS scans rendered through Imaris viewer of single point lesions. Images rotated 90° with respect to the cloud image. The face of the transducer is at the top, with the acoustic propagation going from top to bottom.

Following treatment, gels were trimmed to expose the lesion region and imaged using phase contrast microscopy and open-top light-sheet microscopy (OTLS) for qualitative assessment of lesion morphology. Distinct lesion patterns were observed across pulse regimes (Figure 3). For the 100, 1,000, and 10,000 cycle conditions, lesions contained a contiguous liquefied region surrounded by sparse microscopic damage. These lesions appeared as densely packed, ellipsoid-shaped regions on phase contrast microscopy and were similarly observed in OTLS renderings. On B-mode ultrasound, these treatments produced a hypoechoic region consistent with liquefaction, surrounded by a hyperechoic region corresponding to partial or sparse damage.

In contrast, the 1-cycle and 10-cycle conditions produced sparse, discontinuous damage patterns rather than contiguous liquefaction. Phase contrast microscopy showed microscopic pockets of damage interspersed with intact gel, forming elongated or tubular-like structures. OTLS renderings further confirmed these sparse damage patterns. On B-mode ultrasound, these treatments primarily appeared hyperechoic, without a clearly measurable hypoechoic region. Overall, these findings indicate that short-pulse, high-PRF conditions produced treatment-affected regions but did not generate the contiguous liquefaction observed with pulse durations ≥100 cycles.

### B-mode and SWE assessment of single-point treatments

Treatment response was additionally quantified using SWE and B-mode ultrasound. SWE was used to measure treatment-induced stiffness reduction, whereas B-mode imaging was used to distinguish total treatment-affected volume from fully liquefied volume. Hyperechoic regions were interpreted as sparse or partial damage, while hypoechoic regions were interpreted as fully liquefied tissue based on comparison with microscopy based on our previous characterization of the phantoms^34^. Therefore, total damage volume refers to the combined hyper- and hypoechoic treatment-affected region, whereas hypoechoic volume represents the measurable fully liquefied component.

**Figure 4.**
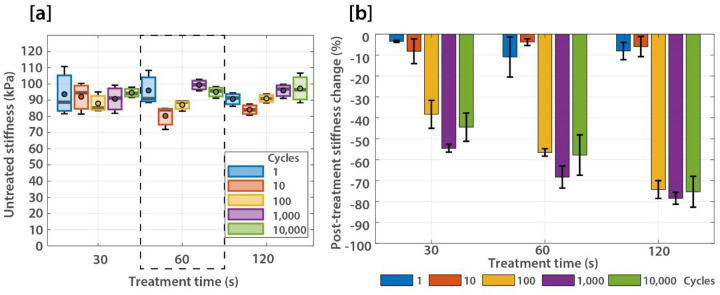
Gel stiffness prior to treatment (a) and change in stiffness in the focal zone post treatment (b). Negative changes indicate a decrease in stiffness from each sample’s initial value prior to treatment

Untreated gel stiffness values were similar across treatments and doses (Figure 4a), with a mean stiffness of 91.7±7.9 (s.d.) kPa. The values ranged from 68.7 to 110.8 kPa, indicating moderate baseline variability across gel preparations and measurement locations. Following treatment, stiffness reduction depended on both pulse parameter and dose (Figure 4b). A two-way ANOVA showed significant main effects of cycle and dose, as well as a significant cycle × dose interaction (p < 0.05), indicating that the dose response differed across pulse conditions. The stiffness decreased with dose for the ≥100-cycle conditions, whereas the 1- and 10-cycle conditions showed minimal stiffness reduction and no consistent dose-dependent trend.

At 120 s, stiffness changes of −74.3 ± 4.3 (s.d.)%, −78.5 ± 1.9 (s.d.)%, and −75.3 ± 7.4 (s.d.)% were observed for the 100-, 1,000 -, and 10,000 -cycle conditions, respectively. These changes were significantly greater than those produced by the 1- and 10-cycle conditions at all doses (Tukey HSD, p < 0.05). In contrast, the maximum stiffness change for the 1- and 10-cycle conditions were only −8.2 ± 5.9 (s.d.)% and −10.9 ± 9.6 (s.d.)%, respectively.

**Figure 5.**
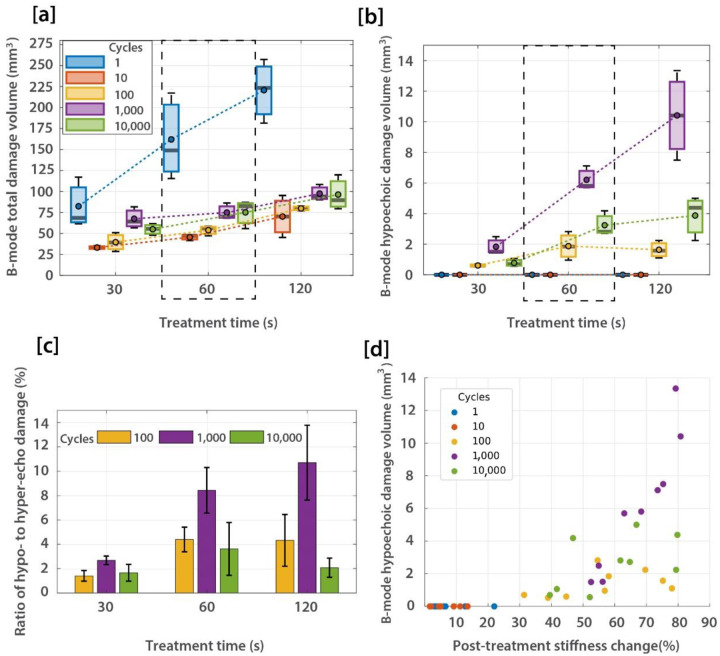
B-Mode results from single point treatments. (a) shows the B-mode total damage volume encompassing hyperechoic and hypoechoic regions. (b) shows B-mode hypoechoic volume only, while (c) displays the ratio of hypo to hyperechoic damage. (d) B-mode hypoechoic volume vs post-treatment reduction in stiffness (%) is displayed.

B-mode-derived volume measurements showed that total damage and fully liquefied volume did not scale together across pulse regimes (Figure 5a,b; Table 1). Total damage volume increased with treatment time across all conditions (Figure 5a). The 1-cycle treatment produced the largest total damage volumes, followed by the 10-cycle condition, but neither generated measurable hypoechoic regions at any dose. This result indicates that the observed damage remained sparse or incomplete rather than fully liquefied.

Hypoechoic volumes were observed only for pulse durations ≥100 cycles (Figure 5b). Among these conditions, the 1,000 -cycle treatment produced the largest mean hypoechoic volumes across treatment times. The hypoechoic-to-total damage ratio further showed that the 1,000 - cycle condition had the highest conversion of total damage into fully liquefied volume (Figure 5c). The relationship between post-treatment stiffness change and hypoechoic volume for single-point treatments is shown in Figure 5d. Conditions that produced measurable hypoechoic volumes were generally associated with larger stiffness change, whereas the 1- and 10-cycle conditions clustered near zero hypoechoic volume with minimal stiffness change. These results suggest that stiffness reduction reflected the presence of localized complete liquefaction, although it did not fully capture differences in liquefied volume across pulse conditions.

Despite differences in liquefaction behavior, total damage volumes among the 100-, 1,000 -, and 10,000 -cycle conditions were not statistically different (Table 2). ANCOVA analysis showed that the slope of total damage volume with dose for the single-cycle condition was significantly different from all other conditions (p < 0.001), whereas no significant differences in slope were observed among the remaining conditions. For fully liquefied volume, the slope for the 1,000 -cycle condition was significantly different from both the 100- and 10,000 -cycle conditions (p < 0.0001). These results indicate that larger total affected volume did not necessarily correspond to more complete liquefaction.

### Open-top light-sheet microscopy assessment of single-point treatment-affected volume

**Figure 6.**
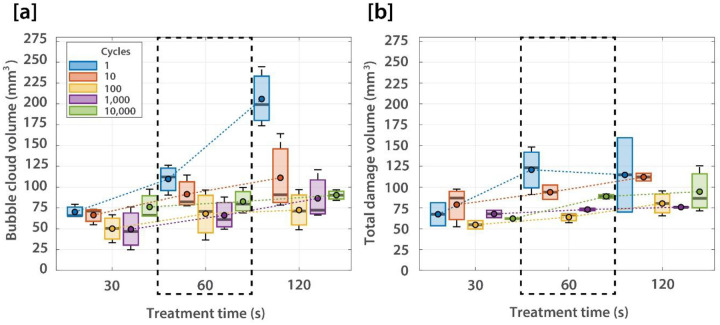
Comparison between bubble and damage volumes by OTLS microscopy. (a) shows total volume measurement from cumulative bubble cloud images for each parameter and dose, (b) shows total damage volume measurement from OTLS scan for each parameter and dose.

OTLS was used to provide a nondestructive 3D assessment of treatment-affected volumes in single-point lesions. OTLS measurements were compared with cumulative bubble cloud volume estimates and B-mode-derived damage volumes to evaluate whether cavitation extent corresponded to measurable structural damage (Figure 6).

For the ≥100-cycle treatments, bubble cloud volume estimates showed good agreement with total damage estimates from both B-mode and OTLS imaging, with no statistically significant differences (paired t-test p>0.05) observed between modalities. This suggests that, for conditions producing more contiguous lesions, cumulative cavitation extent, acoustic imaging, and optical 3D assessment captured similar treatment-affected regions.

For the 10-cycle condition, no significant difference was observed between bubble cloud volume and OTLS-derived damage volume. However, both bubble cloud and OTLS estimates were significantly different from B-mode estimates (p < 0.001 and p < 0.0001, respectively), with average differences of 36.7 ± 7.0 (s.d.) mm^3^ and 49.3 ± 5.5 (s.d.) mm^3^, respectively. This indicates that B-mode underestimated the treatment-affected volume for this sparse damage regime.

For the 1-cycle condition, no significant difference was observed between bubble cloud volume and OTLS-derived damage volume. However, bubble cloud volume differed significantly from B-mode estimates (p < 0.05), with an average difference of −33.5 ± 11.3 (s.d.) mm^3^, indicating overestimation by B-mode. Together, these findings show that imaging-based volume estimates are strongly dependent on lesion morphology and contrast mechanism, particularly under sparse cavitation conditions.

### Lesion morphology after multi-point treatments

**Figure 7.**
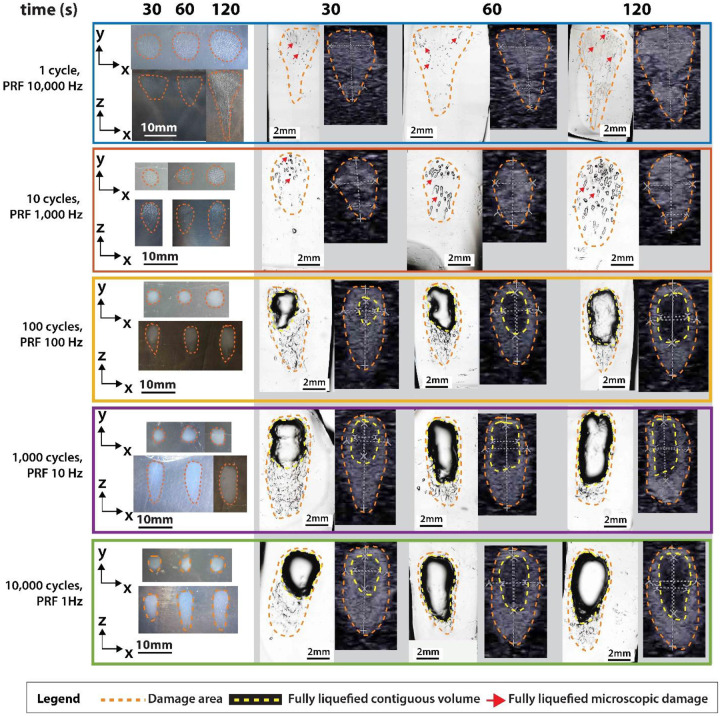
Phase contrast microscopy images and B-Mode imaging for multi-point lesions. 1 and 10-cycle treatments demonstrate sparse damage with hyperechoic regions (orange outline). 100-cycle and greater pulse durations demonstrated by hyperechoic and hypoechoic (yellow outline) regions, consistent with single-point treatments. The face of the transducer is at the top, with the acoustic propagation axis going from top to bottom.

Multi-point treatments were performed to determine whether the lesion patterns observed in single-point exposures persisted during spatially overlapping treatments. Qualitative assessment using phase contrast microscopy showed that the same parameter-dependent trends were maintained in multi-point treatments (Figure 7).

For the ≥100-cycle conditions, multi-point treatments produced widened, densely packed (50% overlap) ellipsoidal lesions with more contiguous liquefaction. These lesions appeared as continuous hypoechoic regions on B-mode imaging with a surrounding hyperechoic boundary, consistent with the single-point observations. In contrast, the 1-cycle and 10-cycle treatments produced sparse and discontinuous damage, with intact gel regions interspersed between damaged zones. These lesions appeared narrower and more elongated and were not clearly resolved as fully liquefied regions on B-mode imaging because of the limited contrast between partially damaged and intact regions.

**Figure 8.**
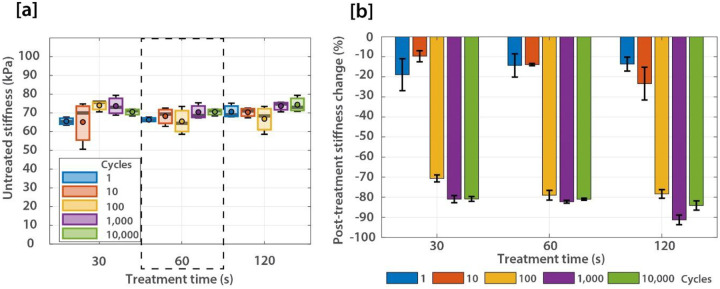
Results of stiffness measurements for multi-point treatments. (a) shows untreated stiffness of the gel used across all treatments. (b) shows post-treatment stiffness change measured as (untreated-treated) %.

The untreated stiffness of gels used for multi-point exposures was consistent across treatment groups (Figure 8a), with a mean value of 69.8 ± 5.4 (s.d.) kPa and no statistically significant differences between groups (p = 0.15). Following treatment, stiffness change depended on both pulse parameter and dose (Figure 8b). A two-way ANOVA showed significant main effects of cycle and dose, as well as a significant cycle × dose interaction (p < 0.05), indicating that dose-dependent stiffness reduction varied across parameter sets.

At 120 s, stiffness change of −23.4 ± 8.2 (s.d.) %, −78.4 ± 2.2 (s.d.) %, −91.3 ± 2.4 (s.d.) %, and −84.1 ± 2.3 (s.d.) % were observed for the 10-, 100-, 1,000 -, and 10,000 -cycle conditions, respectively. The ≥100-cycle conditions produced significantly greater stiffness change than the 1- and 10-cycle conditions at all doses (Tukey HSD, p < 0.05). At 120 s, the 1,000 -cycle condition showed the largest mean stiffness change and was significantly greater than the 100-cycle condition (p < 0.05), while no significant difference was observed between the 1,000 - and 10,000 -cycle conditions. Overall, multi-point treatments showed reduced variability and earlier saturation of stiffness reduction with dose, with ≥100-cycle conditions approaching 80–90% stiffness reduction by 60–120 s.

### B-mode, SWE, and treatment-rate assessment of multi-point treatments

**Figure 9.**
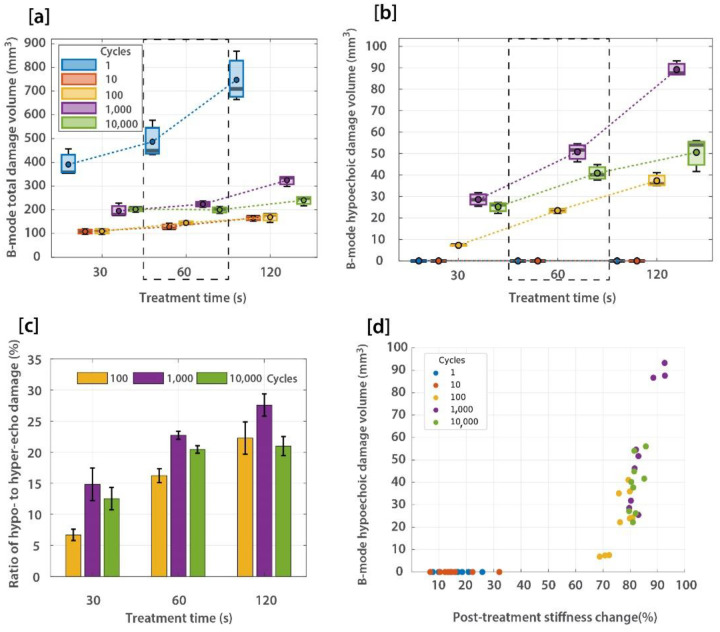
Results of B-Mode imaging for multi-point treatments: (a) shows B-mode total damage volume encompassing hyper and hypoechoic regions vs. dose. (b) shows B-mode hypoechoic volume only vs. dose. (c) displays the ratio of hypo to hyper echo damage in %. (d) B-mode hypoechoic volume vs post-treatment reduction in stiffness (%) is displayed.

B-mode-derived volume measurements from multi-point treatments further confirmed that total damage volume and fully liquefied volume differed across pulse regimes (Figure 9a, b; Table 3). The largest total damage volume was observed for the 1-cycle condition at 120 s, measuring 747.5 ± 106.6 (s.d.) mm^3^, while the lowest volumes at the same dose were observed for the 10-cycle and 100-cycle conditions, measuring 165.1 ± 11.4 (s.d.) mm^3^ and 168.9 ± 19.0 (s.d.) mm^3^, respectively. However, as with single-point treatments, the 1-cycle and 10-cycle conditions did not produce measurable hypoechoic volumes, indicating that these exposures produced sparse or incomplete damage rather than contiguous liquefaction.

Hypoechoic volumes were observed only for pulse durations ≥100 cycles (Figure 9b). Among these conditions, the 1,000 -cycle treatment produced the largest fully liquefied volume at 120 s, measuring 89.2 ± 3.6 (s.d.) mm^3^. The 1,000 -cycle condition also produced the highest hypoechoic-to-total damage ratio at 120 s, 27.6 ± 1.8 (s.d.) %, compared with 22.3 ± 2.6 (s.d.) % for the 100-cycle condition and 21.0 ± 1.5 (s.d.) % for the 10,000 -cycle condition (Figure 9c). These findings indicate that the 1,000 -cycle condition most efficiently converted treatment-affected volume into measurable liquefaction.

The relationship between stiffness reduction and hypoechoic volume is shown in Figure 9d. Most data points associated with larger hypoechoic volumes were located beyond the 75% stiffness-reduction threshold derived from our previous ex vivo prostate study^22^, whereas lower-volume lesions were more dispersed relative to this threshold. This suggests that larger stiffness changes were generally associated with more substantial liquefaction, although stiffness reduction alone did not fully capture differences in liquefied volume across pulse conditions.

ANCOVA analysis of multi-point treatment volumes is summarized in Table 4. For total damage volume, the slope for the single-cycle condition was significantly different from all other conditions (p < 0.0001). Among the remaining conditions, no significant differences in slope were observed, except between the 1,000 - and 10,000 -cycle conditions (p = 0.043). For hypoechoic volume, no significant difference in slope was observed between the 100- and 10,000 -cycle conditions. In contrast, significant differences were observed for the 100–1,000 and 1,000 – 10,000 cycle comparisons (p < 0.0001). The corresponding mean differences in hypoechoic volume were −33.5 ± 14.2 (s.d.) mm^3^ and 17.3 ± 16.8 (s.d.) mm^3^, respectively. These findings further indicate that the 1,000 -cycle condition produced a distinct liquefaction response compared with both shorter and longer pulse-duration regimes.

**Figure 10.**
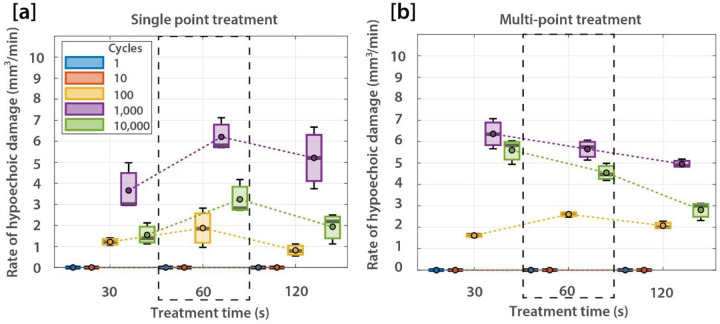
Rate of hypoechoic damage vs. dose for parameters with ≥100 cycles across (a) single point lesion treatments and (b) multi-point lesions.

Volumetric treatment rate was then calculated from hypoechoic volume normalized by treatment time (Figure 10). For single-point treatments, the volumetric treatment rate increased from 30 to 60 s and decreased at 120 s for all ≥100-cycle conditions, with the highest rates observed for the 1,000 -cycle condition (Figure 10a). For multi-point treatments, the volumetric treatment rate showed a different trend for higher-cycle conditions. For the 1,000 - and 10,000 - cycle conditions, the rate decreased with increasing dose, with the maximum values observed at 30 s: 6.4 ± 0.70 (s.d.) mm^3^/min and 5.6 ± 0.58 (s.d.) mm^3^/min, respectively (Figure 10b). In contrast, for the 100-cycle condition, the volumetric rate increased from 30 to 60 s, reaching a maximum of 2.6 ± 0.12 (s.d.) mm^3^/min, and decreased at 120 s.

Comparing single-point and multi-point treatments, the maximum volumetric rate for the 1,000 -cycle condition was similar between the two approaches, occurring at 60 s for single-point treatments and 30 s for multi-point treatments. For the 100- and 10,000 -cycle conditions, higher maximum rates were observed in multi-point treatments compared with single-point treatments. Overall, the 1,000 -cycle condition produced the largest fully liquefied volumes and the highest volumetric treatment rates across both single-point and multi-point treatments.

## Discussion:

Histotripsy has been successfully applied to a range of fibrotic and mechanically robust tissues, including ureteroceles^25^, Peyronie’s plaque^36^, uterine fibroids^18,19^, cholangiocarcinoma^20,37^ and benign prostatic hyperplasia^17,22,23^. Prior studies have demonstrated efficient ablation using both low-PRF, longer-pulse exposures and high-PRF, short-pulse (including single-cycle) regimes^19,22,27^. However, these studies were conducted across different systems and tissue models, making direct comparison challenging. In the present study, pulse duration and PRF were varied systematically at a constant duty cycle within a fibrous tissue-mimicking hydrogel, allowing direct comparison of cavitation behavior and treatment outcome across regimes. The results show that treatment outcome depended significantly on pulse duration and PRF, despite maintaining a constant duty cycle across pulse-parameter sets. The volumetric treatment rates reported here were comparable to those described in prior *ex vivo* fibrotic prostate tissue^22,23^, although direct comparison should be made cautiously because the tissue model and exposure conditions (transducer geometry and operating frequency), slightly differ.

A primary finding was that liquefaction efficiency increased as pulse duration shifted from short-pulse, high-PRF conditions toward longer-pulse, lower-PRF conditions. Among the tested parameters, the highest volumetric treatment rate was observed for 1,000 -cycle pulses delivered at 10 Hz. In our prior *ex vivo* study, efficient ablation was achieved using short pulses (e.g., 20 cycles at 10 Hz)^22^ whereas here substantially longer pulses at the same PRF produced greater treatment efficiency in the fibrous hydrogel model. The common feature in these observations is the efficient ablation is associated with lower PRF, which allows sufficient time for dissolution or redistribution of residual bubbles between pulses. Such processes would enable successive pulses to nucleate bubbles in new locations and prevent shielding of the incident wave, which may be critical to efficient tissue breakdown. Acoustically excited bubbles of relevant sizes have been reported to persist on timescales ranging from milliseconds to seconds^27,38–41^; however, the present study did not directly measure nuclei lifetime or dissolution. Instead, the bubble dynamics data suggest that the effect of persistence depends on its temporal context relative to the pulse structure. Under low-PRF conditions, longer pulse durations were associated with more localized cavitation and more effective liquefaction, whereas higher-PRF conditions were associated with greater persistence, spatial bias, and poorer conversion of damage into fully liquefied regions. These observations are consistent with a cavitation-memory effect, but the underlying nuclei dynamics were not directly measured and remain inferential.

Among the tested conditions, 1,000 -cycle pulses delivered at 10 Hz produced the largest fully liquefied volumes and the highest volumetric treatment rates in both single-point and multi-point treatments. Increasing the pulse duration further to 10,000 cycles at 1 Hz did not improve treatment rate, suggesting that an intermediate optimum duration exists to maximize liquefaction efficiency at this duty cycle, and limiting the memory effect is not the only factor driving efficacy.

Multi-point treatments also produced higher rates than single-point exposures for several ≥100-cycle conditions, consistent with spatial overlap between adjacent focal regions. However, treatment rate did not increase monotonically with treatment duration, indicating that longer exposure times increased total liquefied volume but not necessarily liquefaction rate. Together, these findings indicate that the most efficient condition among those tested was an intermediate pulse-duration/PRF regime rather than either the shortest-pulse/highest-PRF or longest-pulse/lowest-PRF extreme.

A second key finding was that short-pulse, high-PRF conditions did not produce liquefactive zones in this phantom. Short-pulse, high-PRF conditions generated substantial total damage volume but little to no measurable fully liquefied volume, indicating that larger cavitation extent did not translate into effective liquefaction. Longer pulse durations (≥100 cycles) at lower PRFs (≤100 Hz) produced contiguous liquefaction across both single-point and multi-point treatments, whereas short-pulse, high-PRF conditions resulted in sparse and discontinuous damage patterns. These differences were accompanied by changes in cavitation behavior, with higher-PRF conditions showing greater spatial persistence, higher frame-to-frame correlation, and stronger cumulative prefocal bias. This behavior is consistent with cavitation memory and with repeated excitation of residual nuclei at similar locations^27,38–41^. At elevated PRFs (≥1,000 Hz), this persistence may limit further nucleation at the focal region during subsequent pulses. Notably, distinct differences were also observed between the two short-pulse conditions: 1-cycle treatments at 10,000 Hz exhibited stronger prefocal cavitation growth, whereas 10-cycle treatments at 1,000 Hz showed reduced prefocal activity, but still failed to generate effective liquefaction. Together, these results suggest that altered cavitation localization and persistence both contributed to reduced treatment efficiency.

Prior studies by Simon *et al* demonstrated efficient ablation of soft tissue phantoms and uterine fibroid tissue using single-cycle pulses at intermediate PRFs (~1–2.5 kHz)^27,42^. In contrast, the present study found that both 1- and 10-cycle exposures were less effective under the higher-PRF conditions tested here. One limitation of the present study was that the PRF for 1-cycle pulses was necessarily kept much higher than typical for intrinsic threshold histotripsy due to the constraint to keep duty cycle (and energy) constant. This extreme PRF may result in a more extreme form of cavitation memory, as we could not ascertain whether cavitation collapse occurred between pulses in this regime. Nonetheless, this difference highlights that there may be limits to increasing PRF to achieve more rapid ablation under such regimes. However, differences from prior single-cycle studies may reflect not only differences in PRF, but also exposure conditions, tissue or phantom composition, and endpoint definitions. Taken together, these findings demonstrate that treatment outcome does not scale monotonically with duty cycle, PRF, or pulse duration alone, but instead depended on the specific temporal distribution of energy delivery.

In addition to influencing efficiency, cavitation persistence appeared to affect the spatial localization and progression of cavitation activity. The cumulative directionality analysis showed that the 1-cycle and 10-cycle conditions exhibited negative directionality bias, with the 1-cycle condition becoming increasingly prefocally biased with treatment duration. This behavior is consistent with repeated excitation of residual nuclei or persistent cavitation activity on the transducer-facing side of the focal region. Effects such as acoustic shielding^43^ or attenuation of the incident waves from the persistent bubble populations may prevent new nuclei from cavitating, although we could not measure this effect directly in this study. In contrast, the ≥100-cycle conditions showed directionality-bias values closer to zero with increasing treatment duration, with the 1,000- and 10,000-cycle conditions approaching a more balanced or slightly distal cumulative distribution at longer durations. This spatial behavior was associated with more contiguous liquefaction and greater stiffness reduction.

In multi-point treatments, where additional spatial interactions are introduced beyond single-point exposures, greater liquefaction rates and clearer separation between parameter regimes were generally observed. This behavior may be associated with the spatial overlap between adjacent focal regions, particularly under conditions of PRF ≤ 100 Hz and pulse durations ≥ 100 cycles. Repeated excitation across neighboring sites may have enhanced cumulative damage and promoted the conversion of partially damaged regions into contiguous liquefaction.

In addition to using previously validated techniques for lesion characterization, we investigated the use of open-top light-sheet (OTLS) microscopy for volumetric lesion assessment. This approach enables nondestructive, three-dimensional evaluation of damage in the fibrous hydrogel model, providing volumetric measurements rather than two-dimensional projections as with phase contrast microscopy. The optical properties of the double-network hydrogel enabled reflective imaging and allowed visualization of treatment-induced damage throughout the volume, improving interpretation of lesion morphology across pulse regimes. Comparison with B-mode imaging highlighted complementary strengths and limitations of each modality. For treatments with ≥100 cycles, volumetric estimates from OTLS, cumulative cavitation cloud imaging, and B-mode were generally consistent, suggesting that these modalities captured similar treatment-affected regions when lesions were contiguous. However, for sparse cavitation conditions such as 1- and 10-cycle exposures, the modalities diverged. These differences likely reflect their distinct contrast mechanisms: B-mode detects changes in acoustic backscatter, whereas OTLS detects optically scattering structural changes within the hydrogel. Therefore, these comparisons should be interpreted as modality-dependent estimates of treatment-affected volume rather than absolute measurements of damage. Several limitations of the OTLS approach were also observed. Fully liquefied regions were not consistently visualized in some cases, likely because regions with reduced scattering provided limited optical contrast. In addition, distinguishing fully liquefied regions from partially damaged regions was challenging because both could exhibit similar image intensity. As a result, OTLS measurements in this study are best interpreted as estimates of total affected volume rather than strictly liquefied volume. Future work may focus on refining OTLS-based measurements to better distinguish partially damaged and fully liquefied regions and on exploring complementary modalities such as optical coherence tomography.

This study has additional limitations that define directions for future work. Experiments were conducted in fibrous hydrogel phantoms that approximate key mechanical properties but do not fully capture ex vivo or in vivo biological complexity; validation in fibrotic tissues will therefore be important. Additional research is needed to correlate the measurements captured by high-speed photography with data on bubble lifetime and dynamics and confirm interpretation of cavitation data in this study. Differences in baseline stiffness between phantom batches used for single-point and multi-point experiments may also influence direct quantitative comparisons across configurations, such as volumetric ablation rates. Although samples within each set were internally consistent, further standardization is needed for stricter comparison between experiment formats. Finally, the parameter space explored here was limited to selected PRF and pulse-duration combinations at a fixed duty cycle. Broader exploration will be required to fully map regime boundaries and identify optimal treatment windows. In particular, the highest PRF investigated (10,000 Hz) was intentionally used to probe cavitation-persistence effects and is not representative of typical intrinsic-threshold histotripsy exposures. Future work examining PRF and pulse duration independently will elucidate their more explicit roles in fibrous tissue ablation with histotripsy.

## Conclusions:

In this study, we systematically evaluated the effects of pulse duration and pulse repetition frequency (PRF) on cavitation behavior and treatment outcomes in a fibrous tissue-mimicking double-network hydrogel under a constant duty cycle. Longer pulse durations at lower PRFs produced more contiguous liquefaction, greater stiffness reduction, and measurable hypoechoic volumes, with a maximum ablation rate achieved for 1,000 cycle pulses at 10 Hz PRF. Short-pulse, high-PRF exposures produced larger total damage volumes but sparse lesion morphology and little to no measurable fully liquefied volume. Overall, this work provides distinct systematic data for understanding how pulse duration and PRF jointly influence histotripsy efficacy in fibrotic media and offers guidance for selecting parameter regimes for improved liquefaction efficiency.

## Methods:

### Fibrous tissue-mimicking hydrogel phantom

Double-network hydrogel phantoms composed of a 90:10 weight ratio of acrylamide to alginate were selected for these experiments because they provide increased stiffness and toughness compared with conventional stiff but brittle hydrogel phantoms. These properties better represent fibrous tissues observed in conditions such as benign prostatic hyperplasia^23,34,44^ and uterine fibroids^45^.

Acrylamide (40% solution) was polymerized using ammonium persulfate (APS), N,N-methylenebisacrylamide (MBAA), and N,N,N’,N’-tetramethylethylenediamine (TEMED) in fixed proportions, with MBAA prepared as a 1% (w/w) stock solution for accurate dosing. Sodium alginate was dissolved under stirring with intermittent sonication, then combined with acrylamide and de-gassed prior to polymerization. The mixture was cast into molds of approximately 30 mm thickness to fully encompass the focal beam and ensure the treatment represented bulk tissue, UV-cured at 60 °C for 3 h, rinsed, and further crosslinked by soaking in a 1M CaSO_4_·2H_2_O solution to achieve complete ionic crosslinking. These phantoms were fabricated following established protocols, with detailed procedures and additional information available in our previous publication^34,46,47^. To accommodate the increased thickness and improve user safety during preparation, the following modifications were introduced:

- *Crosslinking agent adjustment:* N,N’-Methylenebisacrylamide, the crosslinking agent for polyacrylamide, was originally obtained in powder form. A 1% liquid solution was then prepared, and the required quantity was measured and mixed with the gel formulation, as described in our previous work^34^. However, this was replaced with a commercially available 2% liquid solution (M1533) from Sigma Aldrich Inc. (St. Louis, MO, USA), with corresponding adjustments made to the final volume calculations. For convenience, Table 5 provides the exact quantities required to prepare 200 g of 90:10 acrylamide-alginate gel.
- *Crosslinking time adjustment*: Because these gels were thicker than those used in our previous work, the CaSO_4_·2H_2_O crosslinking time was increased from 48 to 72 hours to ensure complete crosslinking. The gels were then stored in a vacuum-sealed humid chamber for an additional 48 hours to allow the reactions to stabilize and to promote formation of a homogeneous, stiff gel.

### Focused ultrasound system and acoustic calibration

Histotripsy treatments were performed using a 12-element, 1 MHz focused ultrasound transducer with a 65 mm radius of curvature, 90 mm aperture, and f-number of 0.72. This transducer was selected because its low f-number enabled generation of the high focal pressures required across the pulse regimes tested, while its 1 MHz center frequency falls within the range commonly used for histotripsy applications. The transducer had a 28 mm central opening for coaxial mounting of an imaging transducer (3PE, Humanscan, Gyeonggi-do, South Korea), which was connected to a Verasonics system (V-1 Ultrasound Acquisition Platform, Verasonics Inc., Kirkland, WA, USA) and operated in 3 MHz B-mode for treatment guidance. The therapeutic array was driven by a custom Class D amplifier powered by a high-voltage source (TDK Lambda GENH600–1.3) and controlled using an FPGA board (Altera DE0-Nano, Terasic Technology, Dover, DE, USA).

Calibration of transducer output as a function of amplifier voltage was performed using a fiber-optic hydrophone (FOPH2000, RP Acoustics, Stuttgart, Germany). Focal pressure waveforms were recorded for 10–30 μs pulses at increasing input source voltages until cavitation occurred at the fiber tip. Measurements were taken in the steady-state portion of the waveform after the initial ring-up phase. Linear beamwidth measurements were obtained using a needle hydrophone (Precision Acoustics, Dorset, United Kingdom) with a 75 μm active area at a low acoustic output, where nonlinear distortion of the waveform was expected to be negligible and the focal pressures remained well below treatment amplitudes; this corresponded to a 4V DC input voltage for the present system.

**Figure 11.**
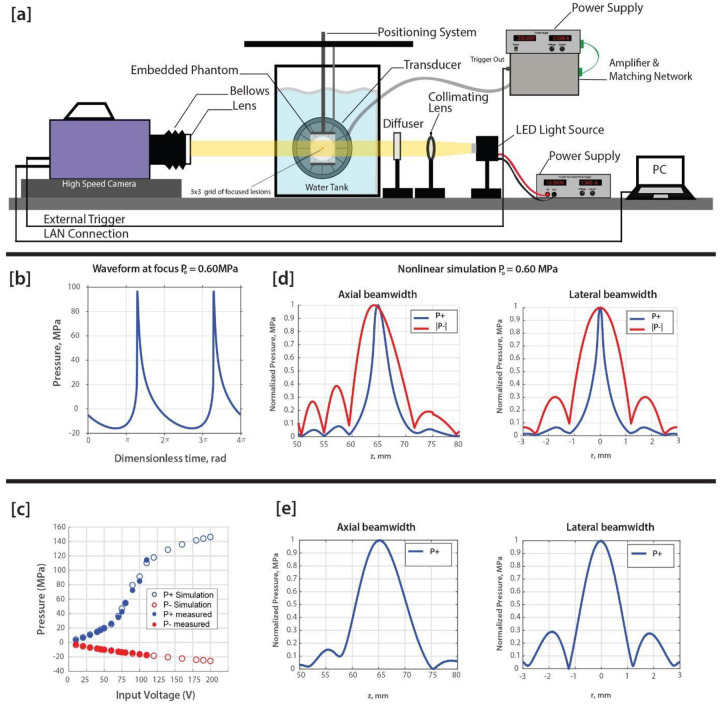
(a) Schematic of the experimental setup for high-speed imaging, including the water tank, transducer, embedded phantom, LED backlighting system (collimating lens and diffuser), high-speed camera, and three-axis positioner. (b) Fiber-optic probe hydrophone (FOPH) waveform measured under representative treatment conditions used for most exposures (except single-cycle conditions), showing near-shock formation. (c) Peak positive and peak negative pressures as a function of DC input voltage, comparing FOPH measurements with nonlinear simulations (HIFU beam) using the empirical calibration described in methods d) Simulated normalized axial and lateral beam profiles under nonlinear propagation, corresponding to a source pressure of 0.60 MPa. (e) Axial and lateral beam profiles measured using a needle hydrophone in the linear regime at low input voltages, used to estimate the −6 dB beamwidth.

Nonlinear simulations were performed using HIFU beam software, which solves the Westervelt equation using a wide-angle parabolic approximation^48^. Simulations were performed using the nominal axisymmetric transducer geometry, including the measured radius of curvature, aperture, and central opening, with a spatially uniform source vibration pattern assumed at the transducer surface. To relate experimental drive voltage to simulation input, an empirical linear source pressure scaling was selected by comparing simulated peak positive and peak negative focal pressures with FOPH measurements over the measurable multi-cycle voltage range from 10 to 110 V (Figure 11c) as follows:

(1) $$P_{0}\left[MPa\right]=0.005V+0.0331$$

where *V* is the DC source voltage. Using this scaling, simulations accurately capture FOPH measurements of the peak positive (R^2^ = 0.993) and peak negative (R^2^ = 0.997) pressures. Consequently, simulations are presumed to provide reasonable estimates of the pressure field at output levels that exceed the range where FOPH measurements were feasible.

For the single-cycle condition, which exceeded the hydrophone measurement range, the calibrated simulation framework was used to estimate focal pressures after accounting for the reduced first-cycle amplitude relative to the steady-state waveform. This correction was estimated empirically from FOPH measurements acquired across multiple drive levels from 10 to 110 V by relating the first-cycle peak and trough amplitudes to the corresponding steady-state waveform amplitudes. The measured first-cycle-to-steady-state relationships were then applied to the simulated steady-state focal pressures at 240 V to estimate the in situ focal pressures for the single-cycle exposure. For the negative-pressure relationship, pressure magnitudes were used for the regression, and the final value is reported using the negative-pressure sign convention.

The theoretical time to boil at representative amplitudes was estimated using weak shock theory:

(2) $$t_{b}=\frac{\Delta TC_{V}6\rho^{2}c^{4}}{\beta f_{0}A_{S}^{3}}$$

where, *ΔT* is the temperature rise from ambient conditions to the boiling temperature (100 °C), *c*_*v*_ is the volumetric heat capacity, *ρ* is the density, *c* is the speed of sound, *β* is the nonlinearity parameter, *f*_0_ is the ultrasound frequency, and *A*_*s*_ is the *in situ* shock amplitude at the focus^6^. This estimate yielded a theoretical boiling time of approximately 1.97 ms for the representative treatment amplitudes. However, pressure amplitude in this study was selected based on empirically measured thresholds on ultrasound imaging rather than boiling threshold. As cavitation and boiling activity are not readily distinguishable using B-mode imaging, all operating conditions are reported relative to appearance of hyperechoic zones on B-mode.

### Pulse parameters and high-speed imaging setup

The transducer was submerged in a tank filled with degassed, deionized water and held in a fixed position. The double-network hydrogel was placed on a fixture mounted to a three-axis motorized positioner with linear slides driven by lead screws and a stepper motor (Bislide and VXM controller, Velmex, Inc., Bloomfield, NY, USA). Depending on the experiment, gels were cast in either cylindrical beakers or rectangular acrylic molds. After curing, gels were removed from the molds and, when required, embedded in thin 1.5% (w/v) UltraPure^™^ agarose (Thermo Fisher Scientific, Waltham, MA, USA) molds to stabilize the sample during high-speed imaging, as described previously.

High-speed imaging was performed using a Photron Fastcam APS-RX camera (Photron, San Diego, CA, USA) paired with a ZEISS Makro-Planar T* 100 mm f/2 ZE prime lens (ZEISS Corporation, Hebron, KY, USA) and an additional bellows. The camera was positioned orthogonal to the acoustic propagation direction. An LED light source driven by a current-controlled DC supply was placed opposite the camera to provide backlighting. A collimating lens and diffuser were used to improve illumination uniformity. The lens aperture was adjusted to provide an approximately 3 mm depth of field. A PC was used to record video using Photron FASTCAM Viewer software and to control the three-axis positioner and FPGA pulse programming through MATLAB^49^.

Five pulse-parameter sets were tested while maintaining a constant duty cycle of 1%. Pulse repetition frequency ranged from 1 to 10,000 Hz, while the number of cycles per pulse was varied inversely from 10,000 to 1 cycle. Treatment durations of 30, 60, and 120 s per point were evaluated based on our previous study examining the dose required to treat *ex vivo* fibrous prostate tissue^22,50^. Specifically, 30s was selected as the minimum exposure duration because it represented the lowest dose at which complete liquefaction was previously observed under longer-pulse conditions. Pulse parameters and camera acquisition settings are summarized in Table 6.

For high-speed imaging analysis, one representative cavitation frame was acquired at 1s intervals throughout treatment. This sampling approach enabled comparison of cavitation evolution across pulse conditions while maintaining a consistent time basis for bubble cloud area, frame-to-frame correlation, cumulative maps, and cumulative directionality bias.

### Treatment protocols

#### Single-point exposures:

Single-point exposures were performed to relate cavitation behavior to lesion morphology, stiffness reduction, and local liquefaction. For each pulse-parameter set and treatment duration, n=3 single-point treatments were performed in the double-network hydrogels. The transducer driving voltage was set to 120% of the threshold at which a consistent cavitation cloud was observed on B-mode imaging during voltage ramping. The camera was triggered with a delay of 44.8 μs relative to the end of the transducer output to account for acoustic propagation time to the focus. The shutter speed was set to 2 μs. One representative frame was acquired at 1s intervals throughout treatment, with the frame timing selected to capture a comparable phase of the cavitation event across parameter sets. The spatial resolution of the recorded images was 33.2 μm/pixel.

#### Multi-point exposures:

Multi-point exposures were performed to determine whether the single-point trends persisted during spatially overlapping treatments and to estimate fully liquefied volume and treatment rate. For each pulse-parameter set and treatment duration, n=3 multi-point treatments were performed on phantoms suspended directly in the fixture without agarose embedding. Each treatment consisted of a 3 × 3 raster-scanned grid with 0.75 mm spacing between adjacent points, corresponding to 50% overlap based on the −6 dB lateral beamwidth. Treatments were performed at 120% of the threshold at which consistent cavitation was observed on B-mode ultrasound. Post-treatment outcomes were evaluated qualitatively and quantitatively using the methods described below.

#### Cavitation image analysis:

Bubble cloud videos acquired during single-point exposures were analyzed in MATLAB after binarization using a custom algorithm described previously^35^. One representative cavitation frame was analyzed for each second of treatment progression. For each processed frame, projected bubble cloud area was calculated from the number of cavitation-positive pixels. Cavitation persistence, used here as an imaging-based indicator of bubble memory effects, was assessed using the Pearson correlation coefficient between successive binarized cavitation frames^34^. Cumulative bubble maps were generated by summing binarized representative frames across each analysis window to track the spatial distribution and recurrence of cavitation activity.

Mean cumulative directionality bias was used to quantify whether cavitation activity accumulated preferentially toward the transducer-facing or distal side of the early cavitation location. For each video, a focal reference line was defined using the early-treatment cavitation centroid. Specifically, the x-coordinate of the cavitation cloud centroid was calculated from the first three usable representative frames, corresponding to approximately the first 3 s of treatment. To reduce sensitivity to noise from a single frame, the reference location was defined as the area-weighted mean x-centroid across these frames. Only frames exceeding a minimum cavitation area threshold of 20 pixels were included in the reference calculation.

For each analysis window, binarized cavitation masks were summed to generate a cumulative cavitation map. Cumulative directionality bias was then calculated by comparing the summed cavitation-positive pixels on the prefocal and post-focal sides of the reference line using:

(3) $$\text{Cumulative directionality bias}=\frac{\left(A_{\text{Post}}-A_{Pre}\right)}{A_{\text{Post}}+A_{Pre}}$$

where *A*_*pre*_ and *A*_*post*_ represent the cumulative cavitation-positive pixel counts on the transducer-facing and distal sides of the reference line, respectively. Negative values indicate preferential prefocal accumulation toward the transducer, positive values indicate preferential post-focal accumulation, and values near zero indicate a more balanced cumulative distribution.

For pooled-window analysis, cumulative directionality bias was calculated within each pulse-duration condition. The 30 s window included the first 30 representative frames from the 30, 60, and 120 s acquisitions; the 60 s window included the first 60 representative frames from the 60 and 120 s acquisitions; and the 120 s window included the full 120 s acquisitions. Data were pooled only within the same pulse-duration condition and were not pooled across different cycle groups.

**Figure 12.**
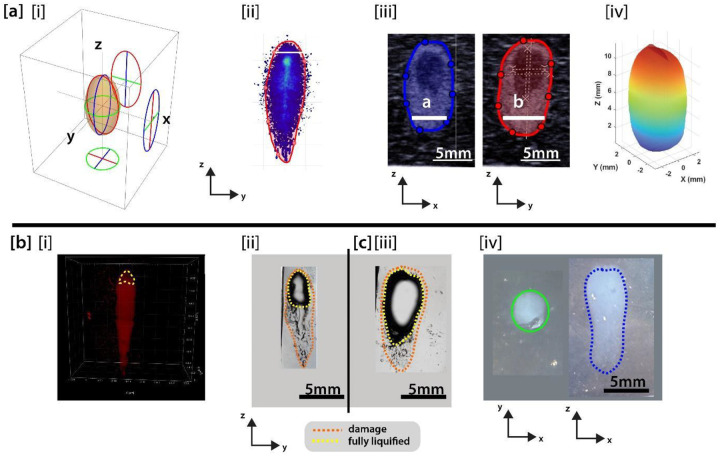
Image-analysis workflow for volume estimation and lesion morphology assessment. (a) Representative masks and geometric approach used to estimate cumulative bubble cloud volume and B-mode-derived treatment volumes. Bubble cloud volume was estimated from cumulative cavitation maps assuming axisymmetry, whereas B-mode volumes were estimated from two orthogonal imaging planes. (b) Representative OTLS rendering generated in Imaris Viewer for three-dimensional visualization of treatment-affected volume. (c) Representative phase contrast and benchtop images of a volumetric treatment lesion, with the fully liquefied region outlined in yellow and total damage region outlined in orange.

Bubble cloud volume estimates were obtained from the cumulative maps at 30, 60, and 120s. Although ellipsoidal geometry has been assumed for bubble cloud volume estimates in prior histotripsy studies^4,26,51,52^, the bubble clouds observed here were variable in shape. Therefore, volume was estimated using a mask fitted to the observed cloud geometry. From this mask (as shown in Figure 12a), individual widths (a) along the Z axis (acoustic) at a single pixel resolution were estimated. Assuming an axisymmetric shape, the bubble cloud volume was calculated using equation 4.

(4) $$V\approx \int \frac{\pi }{4}a{\left(z\right)}^{2}dz,$$

where *dz* is the single-pixel resolution.

#### Lesion morphology imaging:

Lesion morphology was qualitatively assessed using phase contrast microscopy, B-mode ultrasound, and OTLS renderings. We previously demonstrated that phase-contrast microscopy can be used to characterize histotripsy ablation in double-network hydrogels^34^. Damaged and fully liquefied regions exhibit phase variations that appear darker than intact gel, providing contrast to distinguish treatment-induced changes. An example lesion is shown in Figure 12c, along with a corresponding benchtop image of the same lesion. B-mode ultrasound was used to identify hyperechoic and hypoechoic treatment regions, and OTLS datasets were rendered to visualize three-dimensional lesion morphology.

Gels were sectioned through the midplane of the lesion and imaged on a Nikon Eclipse 80i microscope at 2× magnification using a phase contrast filter. A series of images was acquired using NIS-Elements software (Nikon Instruments Inc., Melville, NY, USA) and stitched to visualize the full lesion cross-section. Qualitative assessment was based on: (i) the presence of a contiguous liquefied core, (ii) the extent and distribution of surrounding partially damaged regions, (iii) overall lesion shape and symmetry, and (iv) the presence of sparse or tubular damage patterns indicative of incomplete liquefaction. These features were compared across parameter sets and treatment durations to identify trends in lesion morphology and completeness of liquefaction.

### Quantitative B-mode ultrasound and shear wave elastography analysis

The hybrid gels exhibited sufficient acoustic scattering to enable both B-mode imaging and shear wave elastography (SWE). On B-mode images, fully liquefied regions appeared as continuous hypoechoic zones, whereas partially damaged regions appeared hyperechoic^34^. On SWE, these changes corresponded to reductions in Young’s modulus within treated regions relative to intact gel. B-mode and SWE imaging were performed with the gel suspended in a container filled with degassed, deionized water. A 256-element linear transducer (SL15–4, Supersonic Imagine, Aix-en-Provence, France) connected to the Aixplorer system (bandwidth: 4–15 MHz) was mounted on a custom three-axis manual positioner and aligned with the phantom surface. The transducer was operated in penetration mode. Imaging was performed at the lesion center in the x–z plane (lateral–axial plane). An additional B-mode scan was acquired in the orthogonal y–z plane for volumetric analysis.

#### Stiffness change:

Lesion stiffness was measured using a circular region of interest (ROI) approximately 2 mm in diameter. For treatments exhibiting macroscopic liquefaction, the ROI diameter corresponded to the hypoechoic region observed on B-mode. A reference measurement was obtained from an untreated region, and the percentage change in stiffness between treated and untreated regions was calculated.

#### B-mode Volume estimation:

Two types of volume estimates were made from B-mode images, in a similar fashion to the estimation made from cloud images as described above. The total damage volume that encompassed both hyper- and hypo-echoic regions was estimated from the two B-mode scans taken in the x-z and y-z planes. A mask was drawn around the total damage, as shown in Figure 12a, and the width estimates (“a” along the y-z plane and “b” along the x-z plane) were made at a single pixel resolution along the z-axis. The final volume (*V*) was calculated using the equation

(5) $$V\approx \int \frac{\pi }{4}a\left(z\right)b\left(z\right)\;dz,$$

where *dz* corresponds to the single pixel resolution.

The same approach was applied to estimate the volume of fully liquefied (hypoechoic) regions. The ratio of hypoechoic volume to total damage volume was also calculated.

#### Treatment rate estimation:

Treatment rates were calculated by dividing the hypoechoic volume (fully liquefied volume) by the treatment time and are reported in mm^3^/min.

### Quantitative open-top light-sheet microscopy analysis

An open-top light-sheet (OTLS) microscopy system was investigated as a novel approach to nondestructively evaluate the single point lesions. The orthogonal dual-objective (ODO) arm of the OTLS system was used to capture volumetric datasets of lesions within the phantoms, achieving an isotropic resolution of approximately 4 μm^53^. The phantom gel was placed on a Teflon sample holder designed to match the refractive index of the water/gel (RI = 1.33). Lesions were imaged using a 488 nm laser in scattering mode (i.e., without an emission filter in the detection path). The captured images were stitched and fused using the BigStitcher plugin in ImageJ and saved as a TIFF file with a pixel sampling of 5.8 μm/px^54,55^.

#### OTLS Volume estimation:

The TIFF stack was further processed in FIJI ImageJ, an open-source processing platform^56^ to estimate lesion (total damage) volumes. Once the TIFF stack was loaded into ImageJ, the frames were converted to 8-bit and then thresholded using the “Maximum Entropy Threshold”. This technique maximizes the inter-class entropy. Once thresholded, the total number of pixels representing damage across all the frames was summed and multiplied by the voxel volume (195.1 μm^3^) to obtain the total damage volume. 3D reconstruction of the lesion TIFF stack was done using Imaris viewer (Oxford Instruments, Abingdon, UK) for visualization as shown in Figure 12b.

### Statistical analysis

Statistical analyses were performed using JMP (SAS Institute, Cary, NC, USA) with a significance level of α = 0.05. All tests were two-sided unless otherwise specified. Data are reported as mean ± s.d. unless otherwise noted.

For bubble cloud metrics derived from high-speed imaging, including bubble cloud area and Pearson correlation coefficient, one-way ANOVA was used to assess differences across pulse parameters or treatment duration. The null hypothesis was that group means were equal. When a significant effect was observed (p < 0.05), Tukey’s honestly significant difference (HSD) test was used for post hoc pairwise comparisons. Mean cumulative directionality bias values were summarized as mean ± s.d. across pooled video-window measurements within each pulse-duration and analysis-window group.

For single-point stiffness measurements, two-way ANOVA was performed with pulse parameter (cycles) and treatment duration as fixed factors. The null hypotheses were that (1) mean stiffness reduction was equal across pulse parameters, (2) mean stiffness reduction was equal across treatment durations, and (3) there was no interaction between pulse parameter and treatment duration. When significant effects were identified (p < 0.05), Tukey’s HSD test was used for pairwise comparisons within treatment durations.

Dose-dependent trends in total damage volume and hypoechoic volume for single-point treatments were evaluated using analysis of covariance (ANCOVA), with treatment duration as a continuous variable and pulse parameter (cycles) as a categorical factor. The null hypothesis was that the regression slopes were equal across pulse parameters. The interaction term between pulse parameter and treatment duration was used to assess differences in slope (p < 0.05).

Total damage volume measurements obtained from open-top light-sheet microscopy (OTLS) and B-mode ultrasound were compared with bubble cloud volume estimates using paired t-tests grouped by pulse parameter. The null hypothesis was that the mean volumes measured by each modality were equal (p < 0.05).

For multi-point-based stiffness measurements, two-way ANOVA was performed with pulse parameter (cycles) and treatment duration as fixed factors, using the same null hypotheses and post hoc procedures as described for single-point stiffness measurements. For volumetric outcomes under multi-point treatments, ANCOVA was performed using the same framework as for single-point treatments to evaluate treatment-duration-dependent trends across pulse parameters.

## Figures and Tables

**Table 1.** Total damage and hypoechoic (fully liquefied) volumes from B-mode imaging for single-point treatments across pulse parameters (cycles) and treatment time (s) (mean ± s.d.).

Cycles	Total damage volume (mm^3^)			Hypoechoic (fully liquefied volume) (mm^3^)		
	Dose (s)			Dose (s)		
	30	60	120	30	60	120
1	82.4 ± 30.3	162.0 ± 54.4	220.7 ± 38.0	-	-	-
10	33.3 ± 2.1	45.5 ± 3.5	70.2 ± 25.0	-	-	-
100	39.6 ± 11.1	53.9 ± 5.9	79.8 ± 2.9	0.6 ± 0.1	1.9 ± 0.9	1.6 ± 0.6
1,000	67.5 ± 12.8	74.9 ± 9.9	97.5 ± 9.6	1.8 ± 0.6	6.2 ± 0.8	10.4 ± 2.9
10,000	55.0 ± 7.0	75.1 ± 17.0	96.3 ± 21.0	0.8 ± 0.3	3.2 ± 0.8	3.9 ± 1.5

**Table 2.** Comparison of the slope coefficients (ANCOVA analysis, Parameter (cycle) : Dose (time) interaction) on the single point lesions with volume measurements from B-mode scans.

Reference Parameter (Cycles)	2nd Parameter (Cycles)	Total damage (mm^3^)		Hypoechoic (fully liquefied volume) (mm^3^)	
		t-ratio	p-value	t-ratio	p-value
1	10,000	−3.7	0.0008*	-	-
1	1,000	−4.1	0.0003*	-	-
1	100	−3.7	0.0008*	-	-
1	10	−3.8	0.0006*	-	-
10	10,000	0.12	0.907	-	-
10	1,000	−0.26	0.797	-	-
10	100	0.12	0.902	-	-
100	10,000	−0.01	0.995	1.7	0.102
100	1,000	−0.38	0.704	6.4	<.0001*
1,000	10,000	0.38	0.708	−4.7	<.0001*

* statistically significant results.

**Table 3.** Tabulation of total damage volume and hypoechoic (fully liquefied volume) across parameters and dose measured from B-mode scans (mean±s.d.) for multi-point lesions.

Cycles	Total damage volume (mm^3^)			Hypoechoic (fully liquefied) volume (mm^3^)		
	Dose (s)			Dose (s)		
	30	60	120	30	60	120
1	390.7 ± 57.3	486.4 ± 79.3	747.5 ± 106.6	-	-	-
10	108.3 ± 11.2	128.4 ± 13.0	165.1 ± 11.4	-	-	-
100	109.7 ± 12.1	144.9 ± 7.3	168.9 ± 19.0	7.3 ± 0.4	23.5 ± 1.1	37.4 ± 3.3
1,000	195.5 ± 28.0	223.5 ± 13.7	324.2 ± 22.9	28.6 ± 3.2	50.8 ± 4.3	89.2 ± 3.6
10,000	202.0 ± 10.5	200.0 ± 14.6	240.1 ± 20.5	25.2 ± 2.6	40.9 ± 3.7	50.6 ± 7.8

**Table 4.** Comparison of the slope coefficients (ANCOVA analysis- Parameter (cycle) : Dose (time) interaction) on multi-point lesions with volume measurements from B-mode scans.

Reference Parameter (Cycles)	2nd Parameter (Cycles)	Total damage (mm^3^)		Hypoechoic (fully liquefied) volume (mm^3^)	
		t-ratio	p-value	t-ratio	p-value
1	10,000	−7.4	<.0001*	-	-
1	1,000	−5.3	<.0001*	-	-
1	100	−7.1	<.0001*	-	-
1	10	−7.1	<.0001*	-	-
10	10,000	−0.36	0.725	-	-
10	1,000	1.8	0.090	-	-
10	100	−0.02	0.988	-	-
100	10,000	−0.34	0.736	−1.21	0.233
100	1,000	1.8	0.087	7.7	<.0001*
1,000	10,000	−2.1	0.043*	−8.92	<.0001*

* show statistically significant results.

**Table 5.** Sample weight and volume measurements for preparing 200 g of 90:10 polyacrylamide-alginate gel

Chemical name	Final quantity
De-ionized water	103.96 ml
Acrylamide (40% solution)	63 ml
Sodium alginate	2.8 g
Ammonium persulfate (APS)	From 1% solution, 4.3 ml
N,N-methylenebisacrylamide (MBAA)	From 2% solution 0.76 ml
N,N,N’,N’- tetramethylethylenediamine (TEMED)	91 μL
1-M solution of Calcium sulfate dihydrate	136.1 g in 1L of degassed deionized water

**Table 6.** Pulse parameters and camera acquisition settings.

Parameter No	Pulse repetition frequency (Hz)	Pulse duration (cycles)	Camera frame rate (FPS)	Camera shutter speed (μs)	Dose (s)
1	10,000	1	10,000	2	30,60,120
2	1,000	10	10,000		
3	100	100	10,000		
4	10	1,000	10,000		
5	1	10,000	1,000		

## Data Availability

The datasets generated during and/or analyzed during the current study are available from the corresponding author on reasonable request.
